# Third time recurrent Boerhaave’s syndrome: a case report

**DOI:** 10.1186/s13256-021-02779-5

**Published:** 2021-05-02

**Authors:** Adam Zeyara, Martin Jeremiasen, Oscar Åkesson, Dan Falkenback, Michael Hermansson, Jan Johansson

**Affiliations:** 1 Department of Clinical Sciences in Surgery, Lund University, Lund, Sweden; 2 Department of Surgery, Ystad Hospital, Ystad, Sweden; 3 Division of Esophageal and Gastric Surgery, Department of Surgery, Skåne University Hospital, Lund, Sweden

**Keywords:** Effort esophageal rupture, Boerhaave’s syndrome, Recurrent, Third, Stent, EVAC

## Abstract

**Background:**

Effort rupture of the esophagus or Boerhaave’s syndrome is a rare entity, and prognosis is largely dependent on early diagnosis and treatment. Recurrent effort ruptures are very rare, only reported in a few case reports in English literature. We present a case with a third time effort rupture, and to the best of our knowledge there are no such previous publications. Furthermore, the presented case is also distinct because each episode was treated by different methods, reflecting the pathophysiology of recurrent disease as well as the last decade’s advancements in the management of esophageal perforations in our clinic and globally.

**Case presentation:**

The patient is a 60-year-old White male, suffering from alcohol abuse, mild reflux esophagitis, and a history of effort esophageal ruptures on two previous occasions. He was now admitted to our ward once again because of a third bout of Boerhaave’s syndrome. The first time, 10 years ago, he was managed by thoracotomy and laparotomy with primary repair, and the second time, 5 years ago, by transhiatal mediastinal drainage through a laparotomy and endoscopic stent placement. Now he was successfully managed by endovascular vacuum-assisted closure therapy alone.

**Conclusions:**

Recurrent cases of Boerhaave’s syndrome are very rare, and treatment must be tailored individually. The basic rationale is, however, no different from primary disease: (1) early diagnosis, (2) adequate drainage of extraesophageal contamination, and (3) restoration of esophageal integrity. Recurrent disease is usually contained and exceptionally suitable for primary endoscopic treatment. To cover the full panorama and difficult nature of complex esophageal disease, endoscopic modalities such as stent placement and endovascular vacuum-assisted closure, as well as the capacity for prompt extensive surgical interventions such as esophagectomy, should be readily accessible within every modern esophageal center.

## Background

Boerhaave’s syndrome or effort rupture of the esophagus, is a rare entity, occurring in 3.1 per 1,000,000 and year [1]. It is caused by a sudden increase in intraesophageal pressure combined with negative pressure in the thoracic cavity, such as in severe straining, vomiting, or prolonged coughing [2]. Any part of the esophagus may be affected, but it usually occurs in the distal intrathoracic part, resulting in mediastinitis, and if overlying pleurae are compromised, the disease may extend into the pleural cavities as well. Mortality is high, and if left untreated, Boerhaave’s syndrome unequivocally leads to the demise of afflicted individuals [3]. Albeit clinical features may vary, the classically described presentation is that of a middle-aged man with recent excessive dietary or alcohol intake and repeated episodes of retching and vomiting, typically followed by a sharp pain in the chest and upper abdomen. Diagnosis is usually established by computed tomography (CT) with oral contrast. Endoscopy is often used to confirm the diagnosis.

Principles of management include early diagnosis, adequate drainage of extraesophageal contamination, and restoration of esophageal integrity. In the classical surgical management of Boerhaave’s syndrome, a posterolateral thoracotomy is performed with or without abdominal access depending on the extent of the injury. The lacerated part of the esophagus is exposed, and a primary double-rowed repair is performed. A thorough thoracic irrigation is mandatory, and depending on the extent and nature of the contamination, a decortication of the lung(s) may be necessary. Primary repair by means of thoracoscopy has been reported as well [4]. Also, during the last decade, endoscopic techniques such as endoscopic stent placement and endoscopic vacuum-assisted closure (EVAC) have been gaining territory in this context.

Recurrent effort ruptures are very rare, only reported in a few case reports in English literature [5–8]. We present a case with a third time effort rupture, and to the best of our knowledge there are no such previous publications. Furthermore, the presented case is also distinct because each episode was treated by different methods, reflecting the pathophysiology of recurrent disease as well as the last decade’s advancements in the management of esophageal perforations in our clinic and globally.

## Case presentation

The patient is a 60-year-old White male with a history of alcohol abuse and mild reflux esophagitis. His medications consist of only esomeprazole (40 mg once daily). He is no stranger to our clinic, where he has been treated for Boerhaave’s syndrome on two previous occasions.

**Fig. 1:**
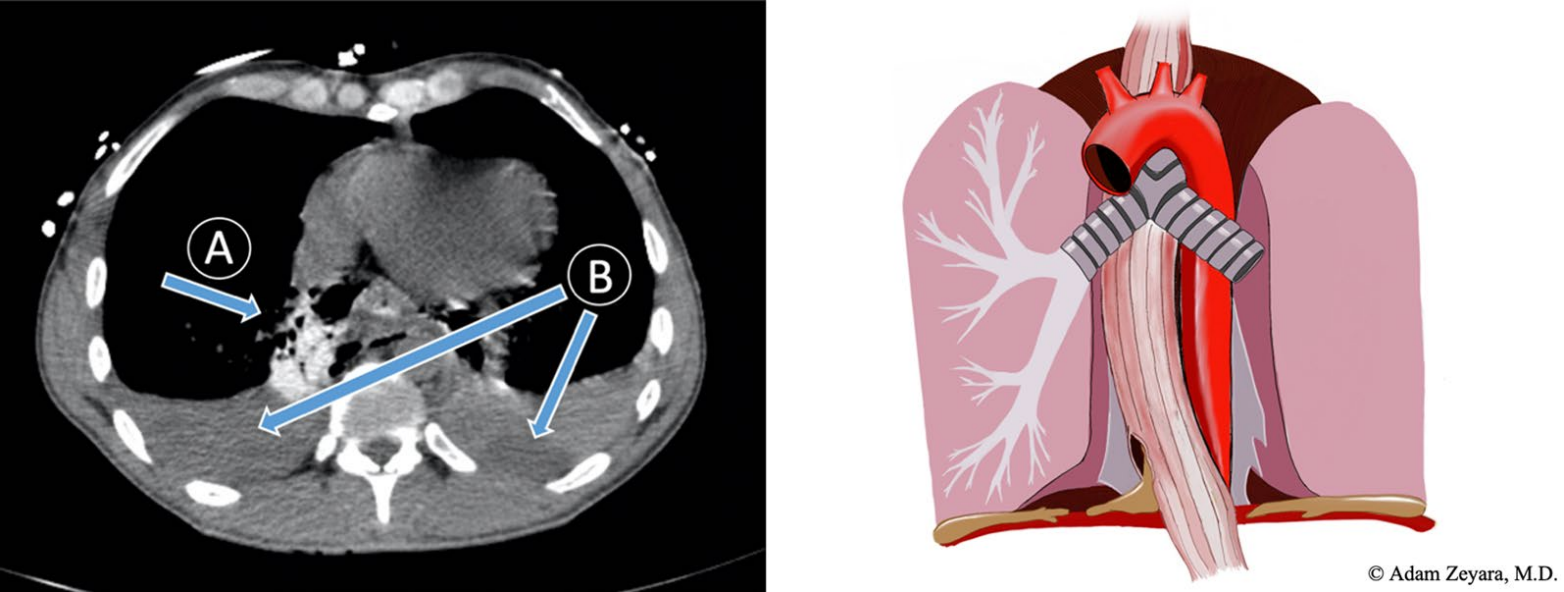
First episode. To the left, a computed tomography-scan of our patient depicting a transverse section of the lower thorax with an esophageal perforation in the native setting and the resultant free extraesophageal extravasation of oral contrast, extensive mediastinal emphysema, and inflammation (**a**), as well as large bilateral pleural effusions (**b**), suggesting that pleurae on both sides have been compromised. To the right, a schematic figure of a distal intrathoracic esophageal rupture in the native setting with free mediastinal contamination and bilateral rupture of the overlying pleurae, resulting in extensive pleural effusions

The first time, 10 years ago, the perforation was large and resulted in a severe mediastinal and pleural contamination (Fig. 1). The diagnosis was confirmed by flexible esophagoscopy. A right-sided posterolateral thoracotomy was then performed. Esophagus was mobilized below the azygos arch down to the hiatal plane, and the perforation was found to be just above the gastroesophageal junction and extending into the abdomen. Having mobilized the esophagus in the thorax, the entire length of the defect could now easily be exposed through an upper midline laparotomy and repaired with a standard double-rowed repair using 4-0 monofilament sutures. A gastrostomy was performed, and a nutrition tube was then placed into the proximal jejunum. Finally, a decortication of the lung was performed, and the thoracic cavity was thoroughly irrigated with warmed saline. External drainage was ensured by large-bore chest drains and abdominal Delbet drains. The immediate postoperative period was uneventful, CT with oral contrast showed no leakage, oral intake was successfully started, and all drains could be removed. However, during a routine follow-up after 1 month, he had developed dysphagia to solid foods, and a stricture in the repaired part of the esophagus was diagnosed. Due to the relatively fresh repair, instead of dilating the stricture, it was treated by placing a temporary fully covered self-expandable metal stent (SEMS). The stent could soon be removed with no residual dysphagia.

**Fig. 2:**
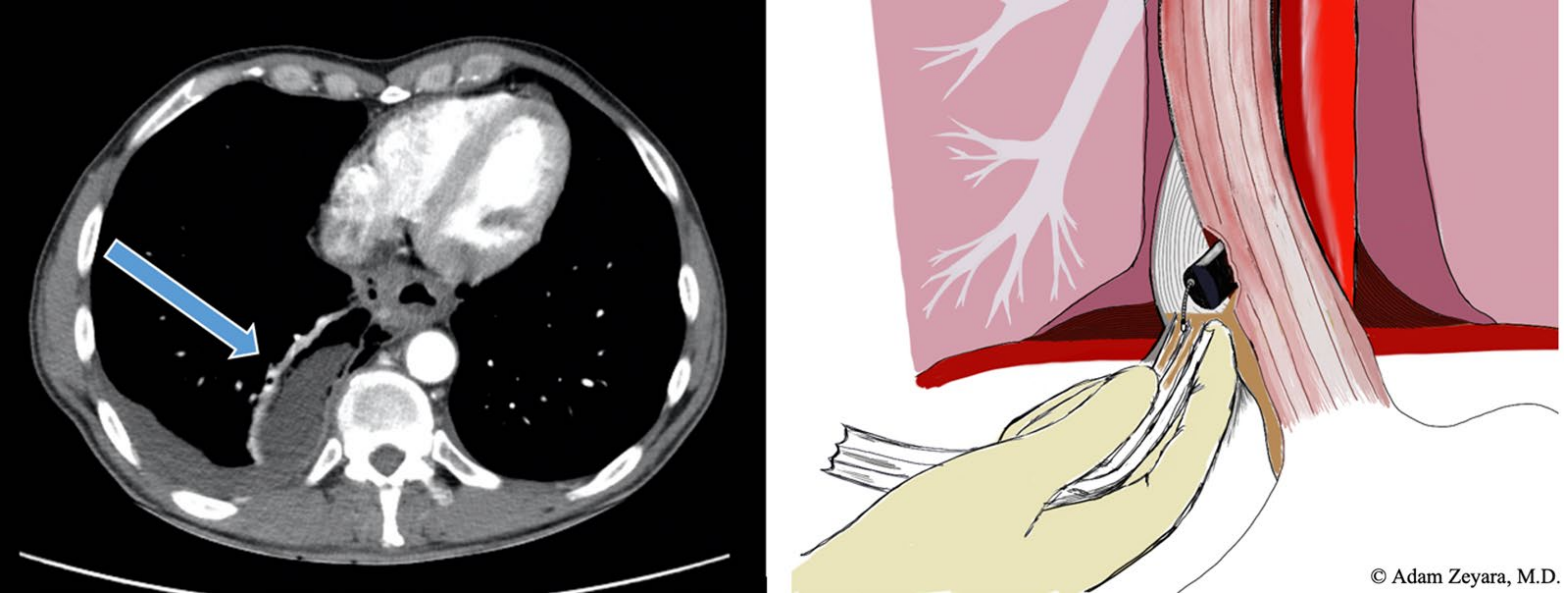
Second episode. To the left, a computed tomography-scan depicting a transverse section of the lower thorax with a large, contained perforation (arrow). Note that there is discrete evidence of mediastinal emphysema and a small pleural effusion on the right side, indicating that the perforation is not entirely walled off. To the right, an illustration of our strategy to ensure adequate drainage. An upper midline laparotomy was performed, and guided by the endoscope, which was advanced extraluminally, a Delbet drain was inserted through the esophageal hiatus and then fixed with precision under visual control by means of endoscopic forceps

The second time, 5 years ago, the extraesophageal extent was also significant, but largely contained, measuring 9 × 5, 5 × 3.5 cm on CT (Fig. 2). A flexible esophagoscopy was performed to confirm the diagnosis. Even though it was contained, the extent of the leakage necessitated robust drainage. Because of the high likelihood of extensive adhesions in the right hemithorax, which would make it very difficult to reach the mediastinum, an upper midline laparotomy approach was chosen instead. Guided by the endoscope, which was advanced extraluminally, a Delbet drain was introduced to the mediastinum through the hiatus and adjusted under visual control by means of endoscopic forceps (Fig. 2). A gastrostomy was performed, and a nutrition tube was placed into the proximal jejunum. Finally, a fully covered SEMS was placed to cover the perforation. A routine CT showed no leakage of oral contrast. The postoperative recovery was initially uneventful, but on postoperative day 10, because of a rising leukocyte count, a control endoscopy was performed. The stent was found to have migrated into the stomach, but the defect was hardly visible anymore. The stent was simply extracted, and nothing more was done. A new CT still showed no evidence of leakage. Oral intake was carefully initiated, and the mediastinal drainage was successively retracted over a period of several weeks. After a total of 2 months, the patient was discharged in good condition. A planned control endoscopy was performed 1 month after discharge, and he was complaining of dysphagia. A stricture was noted in the distal esophagus, and he was treated with balloon dilatations in our polyclinic. After the second dilatation, he was readmitted with a small perforation, which was once again treated with a stent. He received a partially covered stent intended to stay for at least 2 months and then be removed with the so called “stent-in-stent technique” [9]. So, 6 weeks later, a fully covered stent was placed on the inside of the existing one, and after another 2 weeks both stents could be extracted successfully. There was no endoscopic evidence of any residual defect or stricture. During follow-up, he once again developed dysphagia, and underwent regular dilatations in our polyclinic over a period of 12 months. Eventually, his dysphagic symptoms ceased. After another year, he underwent a planned clinical and endoscopic control with no reported dysphagia or visible stricture.

**Fig. 3:**
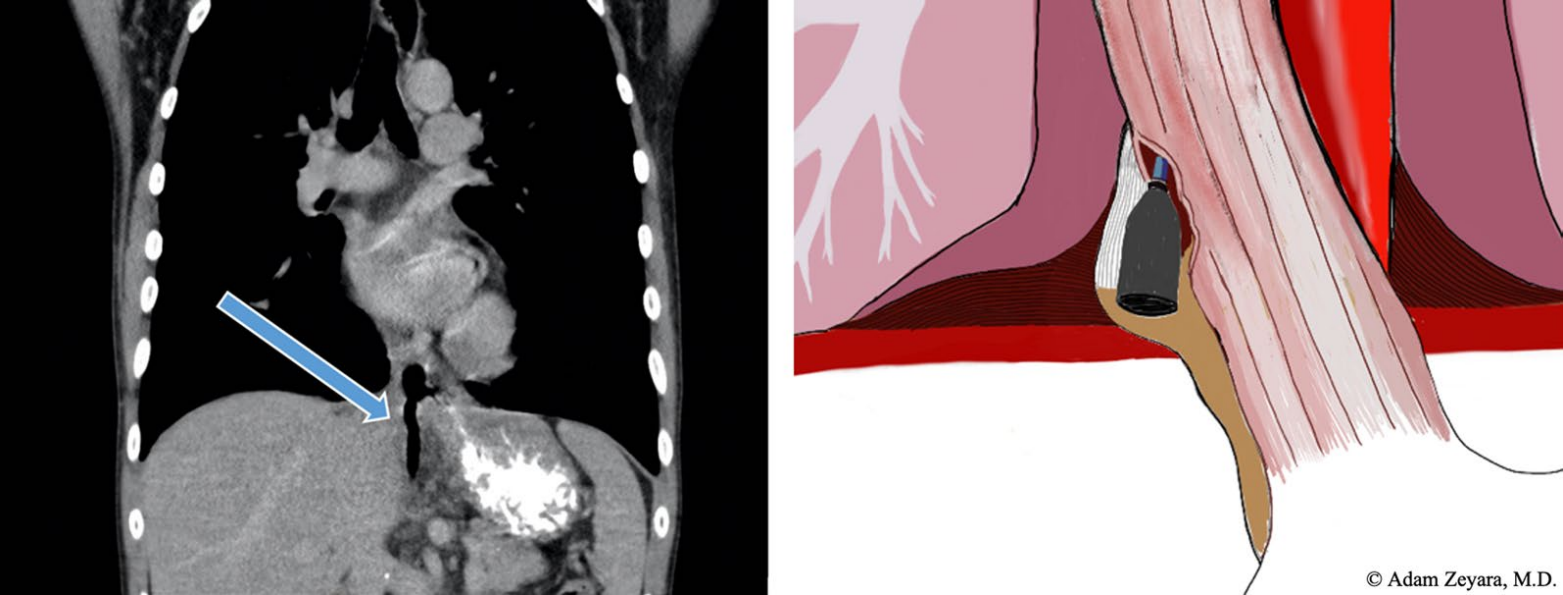
Third episode. To the left, a computed tomography-scan depicting a coronal section of the thorax/upper abdomen with a contained perforation extending caudad along the right paragastric space (arrow). Note that there is no evidence of mediastinal or pleural disease, indicating a completely walled-off perforation. To the right, an illustration of a contained perforation and the extraluminal placement of the Eso-SPONGE system

**Fig. 4:**
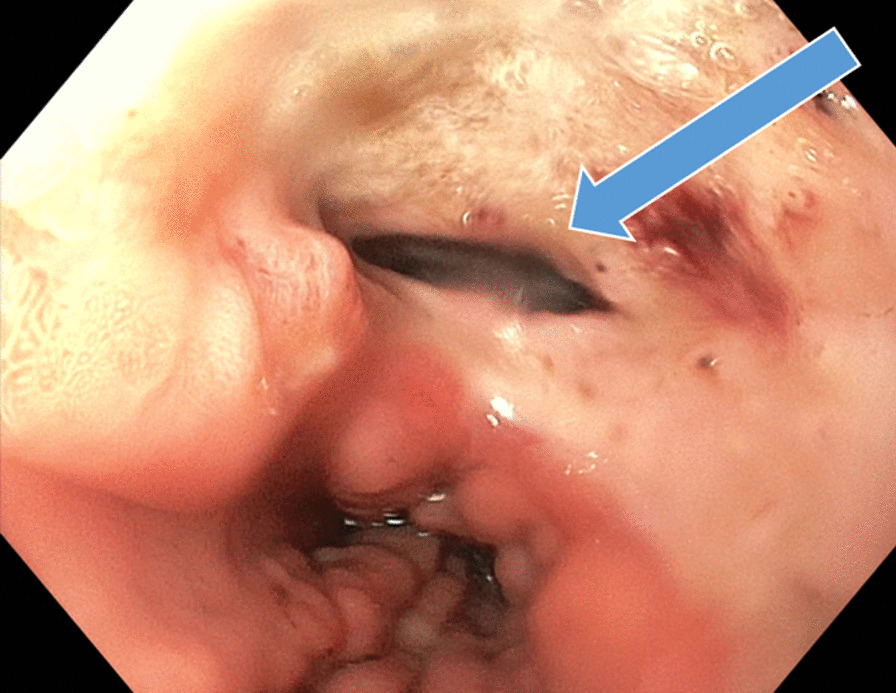
Third episode. Defect visible on endoscopy (arrow)

**Fig. 5:**
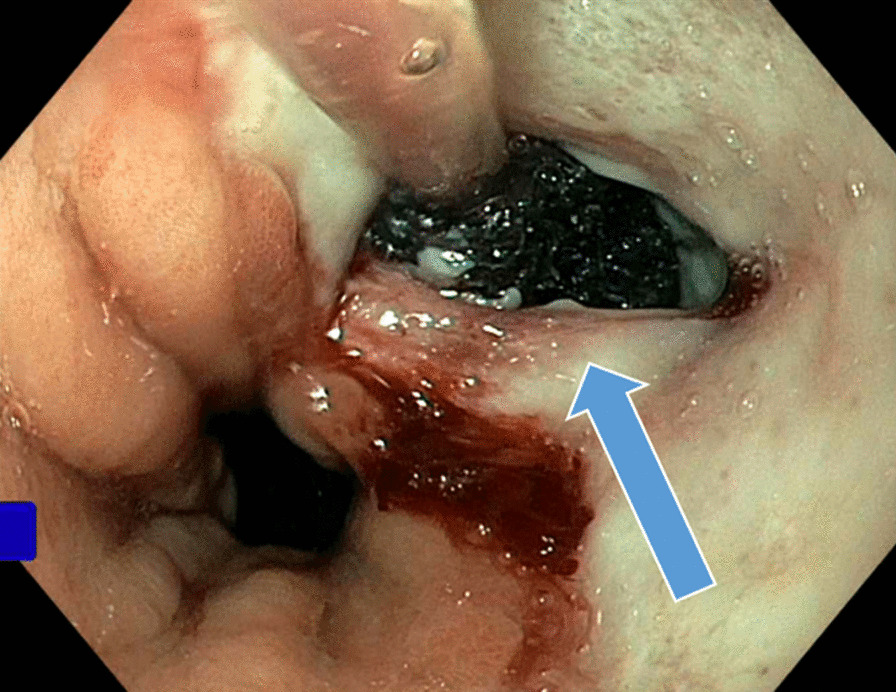
Third episode. Extraluminal Eso-SPONGE (arrow)

**Fig. 6:**
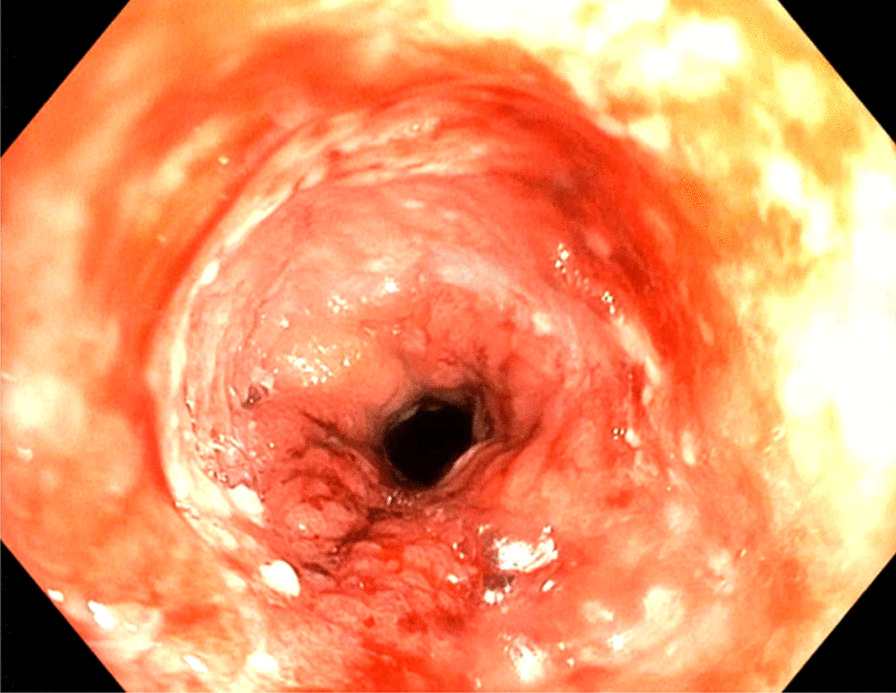
Third episode. After 10 days of Eso-SPONGE treatment, there was no remaining visible defect

**Fig. 7:**
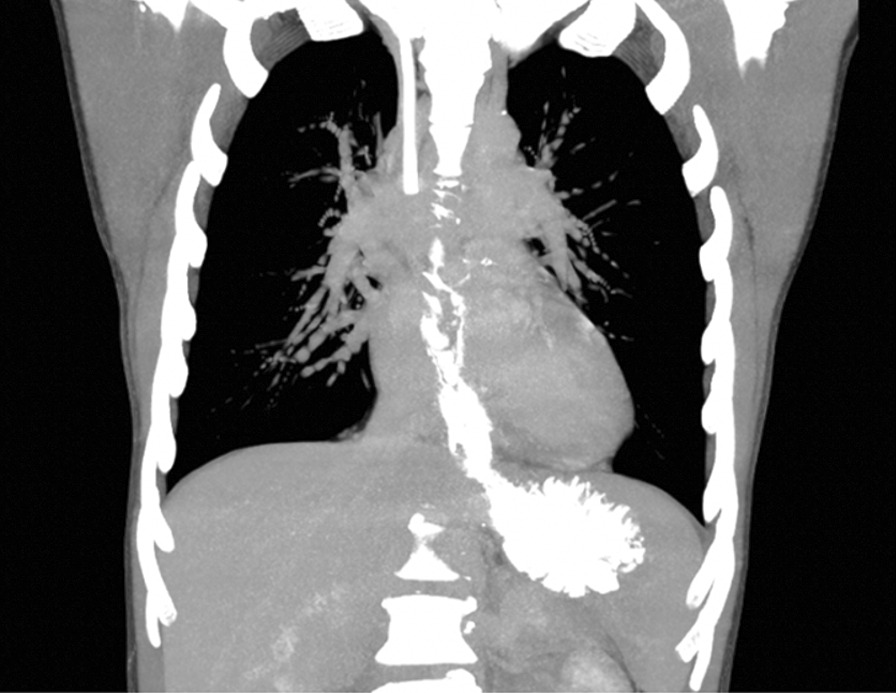
Third episode. No residual cavity or contrast extravasation after Eso-SPONGE treatment

**Fig. 8:**
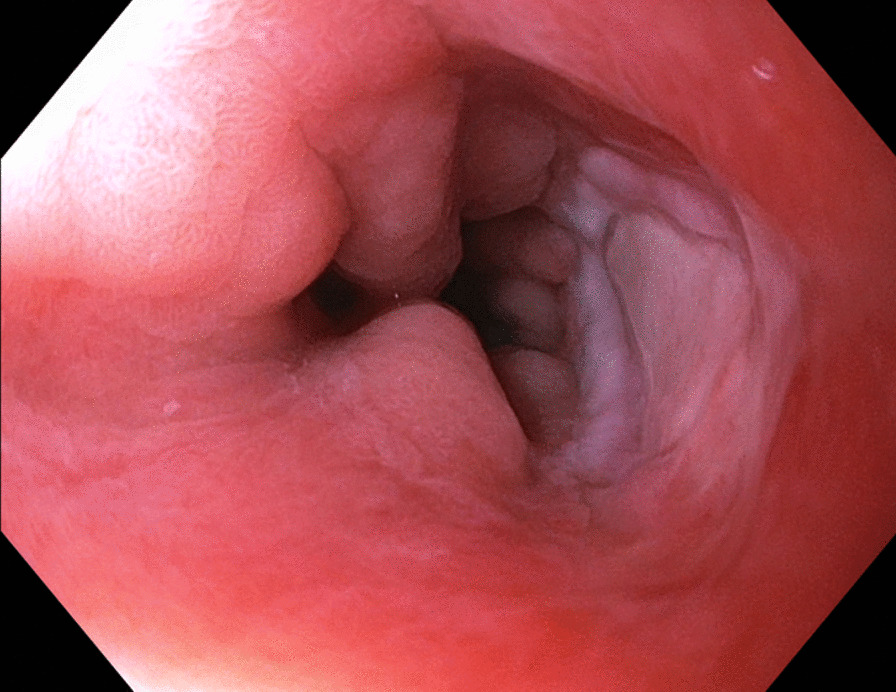
Third episode. Follow-up 3 months after discharge. No evidence of residual defect or stricture

Now, another 5 years later, he was admitted to our ward because of chest pain due to repeated bouts of vomiting after a period of heavy alcohol consumption. CT with oral contrast showed a leakage into a small contained paraesophageal/paragastric cavity, measuring 2.5 × 1 × 5 cm (Fig. 3). His parameters were stable, and leukocyte count was 22.8 × 10^9^/L (normal range 2.5–8.9 × 10^9^/L) and C-reactive protein (CRP) was 9.8 mg/L (normal range < 3 mg/L). A perforation was confirmed by endoscopy (Fig. 4). Because of the contained nature and small extent of the leakage, a minimally invasive approach with EVAC only was attempted. Initially, the sponge was placed extraluminally (Fig. 5) and then successively retracted into the esophagus. After 10 days of EVAC, the defect was no longer visible by endoscopy (Fig. 6). A CT control (Fig. 7) showed no signs of leakage, and oral intake was carefully initiated. Leukocyte count and CRP were practically normal at 9.4 × 10^9^/L and 2.1 g/L, respectively. A planned control endoscopy was performed 3 months after discharge, with no visible defect or stricture (Fig. 8).

## Discussion

A shifting paradigm in the treatment of esophageal perforations is evident, and since the introduction of endoscopic techniques in our center, mortality for all esophageal perforations has decreased significantly [10]. Stent placement can restore esophageal continuity with minimal immediate morbidity but carries a significant risk of leakage and stent migration [11]. EVAC can be used as a single or adjuvant therapy in both recurrent cases and selected primary cases, when primary repair has failed, or as a crossover to stenting [12]. There are also some reports on using the two techniques together, called stent over sponge (SOS) [13].

However, little is known about the long-term outcomes in terms of the quality of the healing process. The rationale in endoscopic stent placement and EVAC in this context is to stimulate granulation and healing by “second intent,” leading to a replacement of defects by fibrosis and adjacent tissues. Surgical repairs are by no means unproblematic either, but intuitively, the end result ought to be more physiologically intact and perhaps more resistant to recurrent disease. It is not known if endoscopically treated patients have a decreased esophageal function or suffer from an increased risk of recurrent disease compared with those undergoing a surgical repair. As the indications for endoscopic treatment even in the primary setting are expanding, long-term outcomes will be needed for esophageal surgeons to properly evaluate the options when choosing a treatment strategy in the emergency setting.

Nevertheless, our case vividly demonstrates the aptness of endoscopic methods in recurrent disease. As demonstrated in the case, the extent and severity of the extraesophageal disease decreased gradually with every episode, largely owing to postoperative adhesions and inflammatory fibrosis, leading to a containment of the extraesophageal extent. This is what makes endoscopic treatment in recurrent disease both suitable and efficient. Attempting to repair a recurrent Boerhaave through a rethoracotomy or relaparotomy would probably be a very difficult surgical venture associated with significant morbidity, especially considering the often very fragile patients suffering from this condition. Should, however, the endoscopic treatment fail, the only feasible surgical option in a third recurrent perforation would probably be an esophagectomy. Although it was not the case in our patient, such a comparison puts things into perspective.

Finally, regardless of which strategy is chosen, surgical or endoscopic, prognosis is still largely dependent on the attainment of adequate drainage of the extraesophageal contamination—as depicted in our case, it might not be that easy in recurrent cases.

## Conclusions

Recurrent cases of effort esophageal rupture are very rare, and treatment must be tailored individually. The basic rationale is, however, no different from primary disease: (1) early diagnosis, (2) adequate drainage of extraesophageal contamination, and (3) restoration of esophageal integrity. Recurrent disease is usually contained and exceptionally suitable for primary endoscopic treatment. To cover the full panorama and difficult nature of complex esophageal disease, endoscopic modalities such as stent placement and EVAC, as well as the capacity for prompt extensive surgical interventions such as esophagectomy, should be readily accessible within every modern esophageal center.

## Data Availability

Not applicable.
